# Music as a non-pharmacological intervention for anxiety during extracorporeal shock wave lithotripsy: a systematic review, meta-analysis, and trial sequential analysis of randomized controlled trials

**DOI:** 10.1007/s11255-026-05266-2

**Published:** 2026-07-21

**Authors:** Jose Arnaldo Shiomi da Cruz, Breno Cordeiro Porto, Joshua Faldini Pinheiro Koren de Lima, Eugênio Vicari Neto, Gustavo Freschi, Diego Pucharelli Resuto, Alex Elton Meller, Rodrigo Afonso da Silva Sardenberg, Ronaldo Soares Maia, Wilson R. Molina

**Affiliations:** 1 Hapvida NotreDame Research Center, São Paulo, SP Brazil; 2 Department of Urology, Federal University of São Paulo Medical School, São Paulo, SP Brazil; 3 Department of Urology, The University of Kansas Health System, Kansas City, KS USA; 4 Department of Urology, Ninth of July Universtiy, São Bernardo do Campo, SP Brazil

**Keywords:** Anxiety, Extracorporeal shock wave lithotripsy, Meta-analysis, Music, Randomized controlled trial, Trial sequential analysis

## Abstract

**Introduction:**

Extracorporeal shock wave lithotripsy (ESWL) is a widely used non-invasive treatment for urinary stones. Nevertheless, procedural noise, discomfort, uncertainty, and repeated shock-wave delivery may cause clinically relevant anxiety. Music has been proposed as an accessible non-pharmacological strategy to improve the patient experience, but previous reviews have reported heterogeneous results and have frequently combined anxiety with pain or audiovisual interventions. We evaluated the association between music exposure and anxiety during ESWL.

**Methods:**

MEDLINE/PubMed, Embase, Scopus, the Cochrane Central Register of Controlled Trials, and Google Scholar were searched from inception to March 2026. Randomized controlled trials comparing music with standard care or no music in adults undergoing ESWL were eligible. Anxiety measured using a Visual Analog Scale (VAS) and the State-Trait Anxiety Inventory (STAI) was analyzed separately. Random-effects meta-analyses were performed using mean differences (MDs) with 95% confidence intervals (CIs). Risk of bias was evaluated using Cochrane RoB 2. Leave-one-out sensitivity analyses, Baujat plots, funnel plots, and trial sequential analysis (TSA) were also performed.

**Results:**

Twelve randomized controlled trials were included in the systematic review. Ten contributed to the VAS analysis and eight to the STAI analysis. Music was associated with lower anxiety measured using VAS (MD –1.01; 95% CI –1.63 to –0.39; *I*^2^ = 80.1%) and STAI (MD –4.46; 95% CI –7.10 to –1.81; *I*^2^ = 91.2%). Leave-one-out analyses showed that the direction of the pooled estimates was not determined by any single study. However, substantial heterogeneity and possible small-study effects were present. The certainty of evidence was rated as very low for both outcomes. Under the pre-specified assumptions, TSA indicated that the cumulative evidence crossed the trial sequential monitoring boundary, although this finding does not address the risk of bias or inconsistency of the underlying studies.

**Conclusion:**

Music was associated with modest reductions in anxiety scores during ESWL and may be considered a feasible non-pharmacological adjunct. Nevertheless, substantial heterogeneity, methodological limitations, variation in music and analgesia protocols, and very low certainty of evidence limit confidence in the magnitude and clinical relevance of the pooled effects. Additional well-designed trials using standardized interventions and clinically meaningful anxiety outcomes remain warranted.

**Supplementary Information:**

The online version contains supplementary material available at https://doi.org/10.1007/s11255-026-05266-2.

## Introduction

Extracorporeal shock wave lithotripsy (ESWL) remains a widely used non-invasive treatment for renal and ureteral stones because it permits stone fragmentation without surgical access and is generally performed in an outpatient setting [1]. Despite these advantages, the procedure may be associated with pain, discomfort, and anxiety, which can adversely affect patient cooperation and tolerance [2].

Anxiety during ESWL is likely multifactorial. Patients may experience anticipatory distress related to pain, uncertainty regarding the procedure, repetitive shock-wave sounds, unfamiliar equipment, and concern about treatment success. Increased anxiety may be accompanied by sympathetic activation, with elevations in heart rate and blood pressure and increased perception of procedural discomfort [2, 3].

Analgesics, sedatives, non-steroidal anti-inflammatory drugs, and opioids are frequently used to improve tolerance during ESWL [4, 5]. However, pharmacological interventions may increase treatment costs, require additional monitoring, prolong recovery, and expose patients to adverse effects. Non-pharmacological interventions that can be implemented without delaying the procedure may therefore provide a useful adjunct to standard peri-procedural care.

Music is one of the most frequently investigated non-pharmacological interventions in procedural medicine. Proposed mechanisms include distraction from unpleasant stimuli, modulation of emotional and limbic responses, attenuation of the physiological stress response, and enhancement of parasympathetic activity [6, 7]. Music may also reduce the salience of lithotripter noise and provide patients with a greater perception of comfort and control.

Previous systematic reviews have examined music and other distraction interventions during ESWL. Wang et al. included five randomized trials and reported reductions in pain and anxiety with music [8]. Hu et al. included 11 trials but combined music and audiovisual distraction and concluded that the evidence was weakened by substantial heterogeneity, small effects, and sensitivity-analysis instability [9]. More recently, Lei et al. evaluated ten randomized trials but focused primarily on pain control rather than anxiety-specific outcomes [10].

The present review differs from previous studies by providing an updated search through March 2026, focusing specifically on music-based interventions and anxiety outcomes, and analyzing VAS and STAI results separately. We also applied RoB 2, influence and heterogeneity diagnostics, and TSA to evaluate the methodological credibility, certainty, and potential risk of random error in the accumulated evidence.

## Materials and methods

### Protocol and reporting

This systematic review and meta-analysis was conducted in accordance with the Cochrane Handbook for Systematic Reviews of Interventions and reported according to the Preferred Reporting Items for Systematic Reviews and Meta-Analyses 2020 statement [11, 12]. The protocol was prospectively registered in PROSPERO under registration number CRD420261374706.

### Search strategy

A comprehensive electronic search was conducted in MEDLINE/PubMed, Embase, Scopus, the Cochrane Central Register of Controlled Trials, and Google Scholar from database inception to March 2026.

The search strategy combined controlled vocabulary and free-text terms related to ESWL, music, and anxiety. The principal search structure was: (“extracorporeal shock wave lithotripsy” OR “extracorporeal shockwave lithotripsy” OR ESWL OR SWL) AND (music OR “music therapy” OR “music intervention” OR “auditory stimulation”) AND (anxiety OR stress OR distress OR “State-Trait Anxiety Inventory” OR STAI OR “visual analog scale” OR VAS).

Reference lists of eligible studies and relevant systematic reviews were also manually screened to identify additional potentially eligible trials.

### Eligibility criteria

Studies were eligible when they met all the following criteria: (1) randomized controlled trial; (2) adult patients undergoing ESWL for renal or ureteral stones; (3) evaluation of a music-based intervention administered before or during ESWL; (4) comparison with standard care, no music, or an otherwise equivalent control condition; and (5) reporting of an anxiety-related outcome.

For multi-arm trials, only the eligible music and control groups were included. Trials evaluating combined interventions were eligible when data from a music-only group and an appropriate control group could be independently extracted.

Studies were excluded if they were non-randomized, involved pediatric populations, did not include an eligible control group, did not report anxiety outcomes, or were conference abstracts, case reports, narrative reviews, or systematic reviews.

### Outcomes

The primary outcomes were post-intervention anxiety scores measured using a VAS for anxiety and the state-anxiety component of the STAI.

VAS and STAI results were analyzed separately because the instruments have different ranges and psychometric properties. When studies reported multiple post-intervention measurements, the assessment obtained immediately after ESWL was prioritized.

VAS results were harmonized to a 0–10 scale before pooling when necessary. When trials contained more than one eligible music group, intervention groups were combined or otherwise incorporated without double-counting the shared control group, in accordance with Cochrane recommendations.

### Study selection

After duplicate removal, two reviewers independently screened titles and abstracts. Potentially eligible articles were retrieved for full-text assessment. The same reviewers independently evaluated the full texts against the eligibility criteria.

Disagreements were resolved through discussion and, when necessary, consultation with a third reviewer. No automated screening or machine-learning tools were used.

### Data extraction

Two reviewers independently extracted data using a predefined form. The following information was collected: author and year; country and institution; study design; sample size; age and sex distribution; stone size and location; type and selection of music; mode and timing of music delivery; use of headphones or noise-canceling headphones; concomitant analgesia or sedation; lithotripter type; anxiety instrument; post-intervention means and standard deviations; and information required for risk-of-bias assessment.

Discrepancies were resolved by consensus or consultation with a third reviewer.

### Risk-of-bias assessment

Risk of bias was assessed independently by two reviewers using Version 2 of the Cochrane Risk of Bias tool for randomized trials (RoB 2) [13]. Each study was classified as having low risk of bias, some concerns, or high risk of bias. Disagreements were resolved by consensus.

### Statistical analysis

Continuous outcomes were summarized using MDs with 95% CIs. Separate meta-analyses were conducted for VAS and STAI because all studies within each analysis used a common instrument or a harmonized version of the same scale.

A random-effects model was selected because clinical and methodological heterogeneity was anticipated. Between-study heterogeneity was evaluated using Cochran’s Q test, the I^2^ statistic, and the between-study variance. I^2^ was interpreted in conjunction with the magnitude, direction, and precision of individual study effects rather than using a rigid threshold alone.

Leave-one-out sensitivity analyses were performed by sequentially removing each study and recalculating the pooled estimate. Baujat plots were used to identify studies contributing disproportionately to heterogeneity or the pooled effect.

Potential sources of heterogeneity included: patient-selected versus investigator-selected music; fixed classical or traditional music versus unrestricted music choice; headphones versus ambient speakers; noise-canceling versus conventional headphones; intervention before versus during ESWL; differences in analgesia or sedation; and methodological quality.

Because the number of studies within most categories was small and several characteristics overlapped, formal subgroup estimates would have been underpowered and potentially misleading. These characteristics were therefore examined descriptively, and the inability to conduct reliable subgroup meta-analysis was considered a limitation.

Funnel plots were visually inspected for possible small-study effects. Egger’s regression test was considered; however, it was not emphasized because only ten studies were available for VAS and eight for STAI, while heterogeneity was substantial. Under these conditions, asymmetry tests have limited power and may be confounded by genuine clinical heterogeneity [14]. All conventional meta-analyses were performed using R and RStudio Software.

### Trial sequential analysis

TSA was conducted separately for VAS and STAI to evaluate whether the accumulated evidence was sufficiently large to reduce the risk of random errors caused by sparse data and repeated significance testing [15, 16].

A two-sided type I error of 5% and statistical power of 80% were used. Diversity-adjusted required information sizes were estimated using a random-effects framework.

## Results

### Study selection and characteristics

**Fig. 1:**
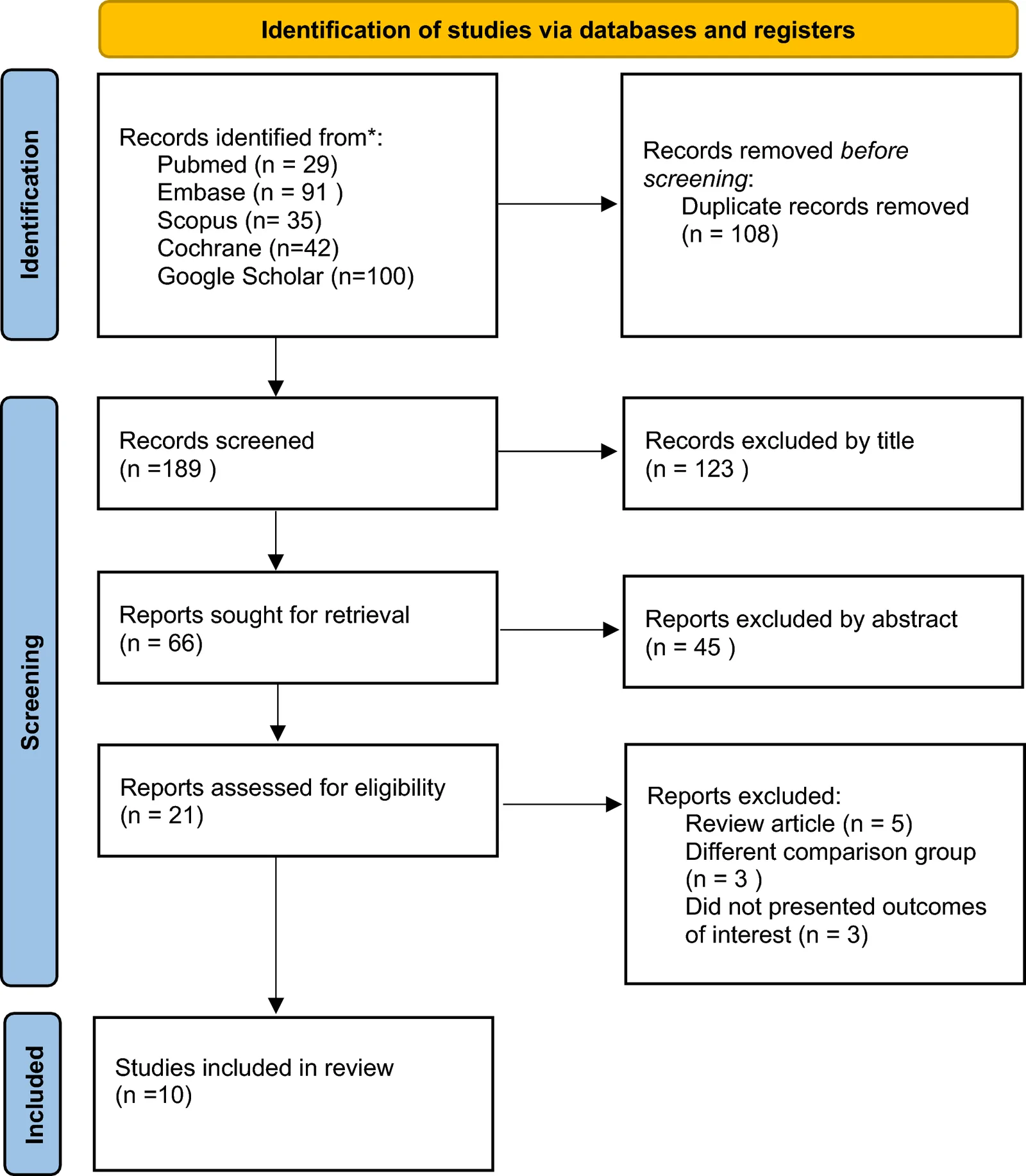
Prisma flow diagram of the study.

The database search identified 297 records: 29 from PubMed, 91 from Embase, 35 from Scopus, 42 from the Cochrane Central Register of Controlled Trials, and 100 from Google Scholar. After removal of 108 duplicate records, 189 records underwent title and abstract screening. Of these, 168 were excluded, and 21 full-text reports were assessed for eligibility. Nine reports were excluded after full-text assessment, resulting in twelve randomized controlled trials being included in the systematic review [17–28]. The study selection process is presented in Fig. 1.

Ten studies contributed data to the quantitative synthesis of anxiety measured using a Visual Analog Scale (VAS), whereas eight studies reported anxiety outcomes using the State-Trait Anxiety Inventory (STAI).

The included studies were published between 1998 and 2022 and comprised 1143 participants in the eligible music and control groups, including 585 participants allocated to music and 558 allocated to control conditions. Overall, 714 participants (62.5%) were male. The mean age of participants ranged from approximately 25–53 years.

The trials were conducted in Colombia, Thailand, Turkey, Spain, Australia, and Iran. The included populations varied regarding stone location and size, lithotripter technology, and peri-procedural management. Eight studies allowed patients to select or indicate their preferred music, whereas four used investigator-selected classical, instrumental, or traditional music. Music was delivered through headphones or headsets in 11 studies and through speakers in 1 study. Analgesic and sedative protocols also varied substantially, ranging from no routine pharmacological analgesia to combinations of non-steroidal anti-inflammatory drugs, opioids, sedatives, and patient-controlled analgesia.

A comprehensive summary of the baseline demographic and clinical characteristics of the included studies is provided in Table 1. Details regarding music selection, mode of delivery, concomitant analgesia, and lithotripter type are presented in Table 2.

**Table 1:** Baseline characteristics of included studies and patient populations

Study	Year	Country	Institution	Design	Music_n	Music_age	Music_male_n	Control_n	Control_age	Control_male_n	Stone_size_mm	Stone_location
Cepeda	1998	Colombia	San Ignacio Hospital, Bogota	Prospective blinded RCT	97	40.7 ± 12.1	48	96	41.0 ± 11.4	47	< 10 to > 20	Renal
Wongwanakul	2015	Thailand	Chiang Mai University Hospital	RCT	28	24.55 ± 4.26	18	29	24.70 ± 4.06	15	NR	NR
Karalar	2016	Turkey	Afyon Kocatepe University, Afyonkarahisar	Prospective RCT (3 arms)	57	47.8 ± 13.7	35	32	48.2 ± 15.5	19	11.8 ± 4.5	Renal calculi
Cakmak	2017	Turkey	Tepecik Training Hospital, Izmir	RCT	95	42.5 ± 12.7	66	105	43.3 ± 12.8	71	NR	Kidney 59%, ureter 41%
Ordaz	2017	Spain	Hospital Univ. La Fe, Valencia	Blind RCT	42	52.6 ± 12.4	23	53	52 ± 13.7	37	NR	Renal 73.7%, ureteral 26.3%
Gezginci	2018	Turkey	Gulhane Military Medical Academy, Ankara	RCT (3 arms)	40	NR	35	40	NR	37	NR	Kidney and ureter
Cift	2020	Turkey	Adiyaman University, Adiyaman	Single-blind RCT (5 arms)	30	36.4 ± 10.2	22	30	38.4 ± 13.9	16	12.0 (median)	Pelvis/UP junction
Khoury	2020	Australia	Casey Hospital, Melbourne	RCT (3 arms)	30	51.5 ± 17.4	21	31	52.6 ± 16.1	22	7.6 ± 2.5	75% renal, 25% ureteral
Dogan	2021	Turkey	Sancaktepe Hospital, Istanbul	RCT (3 arms)	52	44.9 ± 9.4	32	28	48.3 ± 7.9	18	10.4 ± 2.5	Renal/ureteral
Bozkurt	2022	Turkey	Basaksehir Cam & Sakura Hospital, Istanbul	RCT	50	41.78 ± 11.23	31	50	43.24 ± 10.61	29	10.19 ± 3.84	Pelvis 66%, upper ureter 34%
Bueno-Serrano	2022	Spain	Hospital Fundacion Jimenez Diaz, Madrid	RCT (4 arms)	20	52.8 ± 11.7	12	20	52.1 ± 13.8	9	9.4 ± 3.6	LCG 30%, MCG 35%, UCG 35%
Rohi Ganji	2022	Iran	Imam Reza Hospital, Kermanshah	RCT (4 arms)	44	42.8 ± 12.2	24	44	44.0 ± 13.5	27	NR	Pelvis, calyx, ureteral

**Table 2:** Characteristics of music interventions and peri-procedural management across included studies

Study	Music_type	Music_delivery	Analgesia	Lithotripter
Cepeda	Classical, Caribbean, soft rock, jazz (patient selected)	Headphones	Morphine 10 mg SC + ketorolac 30 mg IV + alfentanil PCA	Tripter X1 (electrohydraulic)
Wongwanakul	Classical music	Headphones	NR	NR
Karalar	Turkish classical (ney)	Headphones (NCH + non-NCH)	Diclofenac 75 mg IM	Elmed Multimed Classic (electrohydraulic)
Cakmak	Turkish folk, classical, popular, relaxing (patient selected)	Headphones	NR	EMD E-1000 (electrohydraulic)
Ordaz	Classical, chill out, pop, jazz (patient selected)	Circumaural headphones	Pethidine IV	Siemens Lithoskop (electromagnetic)
Gezginci	Turkish folk, art, pop, rock, classical (patient selected)	Headset	None	Siemens Lithoskop (electromagnetic)
Cift	Self-selected music	Headphones	None	Siemens ModularisVario (electromagnetic)
Khoury	Self-selected (Apple iPad + NCH)	iPad + NCH	NSAID suppository + IM fentanyl PRN	Storz SLK (electromagnetic)
Dogan	Instrumental classical music	Headphones	Diclofenac 75 mg IM + fentanyl IV PRN	PCK V-5 Stonelith (electrohydraulic)
Bozkurt	Patient preferred (no headphones)	Speakers (room)	None	Elmed Multimed EM (electromagnetic)
Bueno-Serrano	Patient’s choice	Headphones	Midazolam 1 mg + fentanyl 0.05mg + dexketoprofen 50 mg IV (all groups)	Storz Modulith SLX-F2 (electromagnetic)
Rohi Ganji	Soft traditional Iranian music (Golnoosh)	MP3 + headphones	NR	Storz SLX 2001 (electromagnetic)

### Meta-analysis

#### VAS (Visual Analog Scale)

**Fig. 2:**
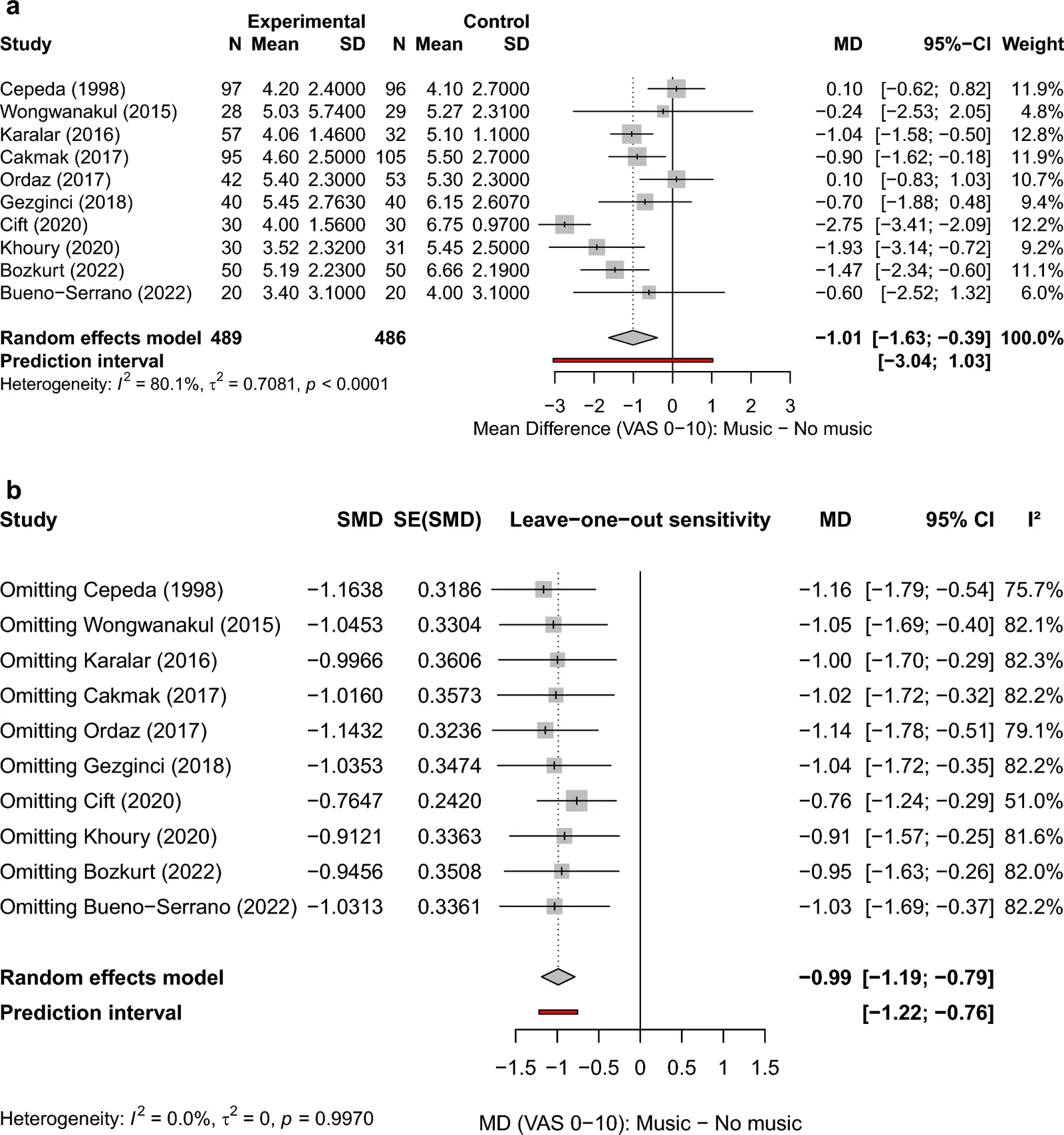

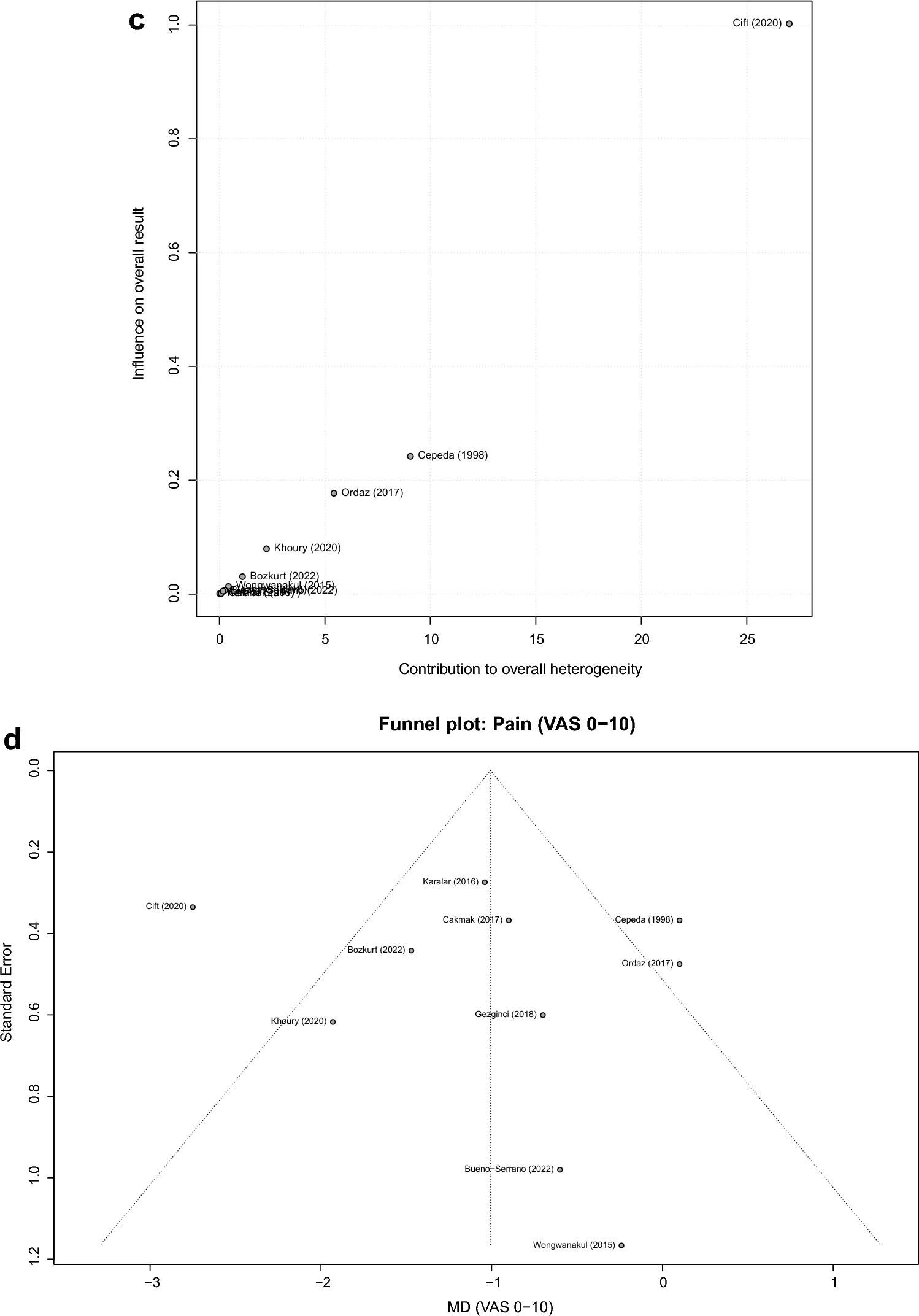
(a) Forest plot of pain on Visual Analog Scale comparing Music versus Control groups (VAS), (b) Leave-one out sensitivity analysis for heterogeneity assessment, (c)Baujat plot was to identify studies contributing most strongly to the overall heterogeneity and pooled effect (d) Funnel plot assessing publication bias among the included studies.

Ten studies were included in the meta-analysis of anxiety measured using VAS. The pooled analysis demonstrated that music was associated with a statistically significant reduction in anxiety compared with the control condition (MD –1.01; 95% CI –1.63 to –0.39; *p* < 0.01). Substantial heterogeneity was observed across studies (*I*^2^ = 80.1%) (Fig. 2a).

To evaluate the influence of individual studies on the pooled estimate, a leave-one-out sensitivity analysis was performed (Fig. 2b). The pooled effect estimates ranged from –0.76 to –1.16 and remained statistically significant after the sequential exclusion of each study. These findings indicate that the direction of the pooled association was not determined by any single study. Nevertheless, substantial residual heterogeneity remained across most iterations.

A Baujat plot was constructed to identify studies contributing most strongly to the overall heterogeneity and pooled effect (Fig. 2c). Although certain studies made greater contributions to heterogeneity, no individual study fully accounted for the observed variability.

The funnel plot showed possible asymmetry, which may indicate small-study effects (Fig. 2d). However, this finding should be interpreted cautiously because funnel plot asymmetry may also result from substantial clinical and methodological heterogeneity. Moreover, with only ten studies, statistical tests of asymmetry have limited reliability.

#### STAI (State-Trait Anxiety Inventory)

**Fig. 3:**
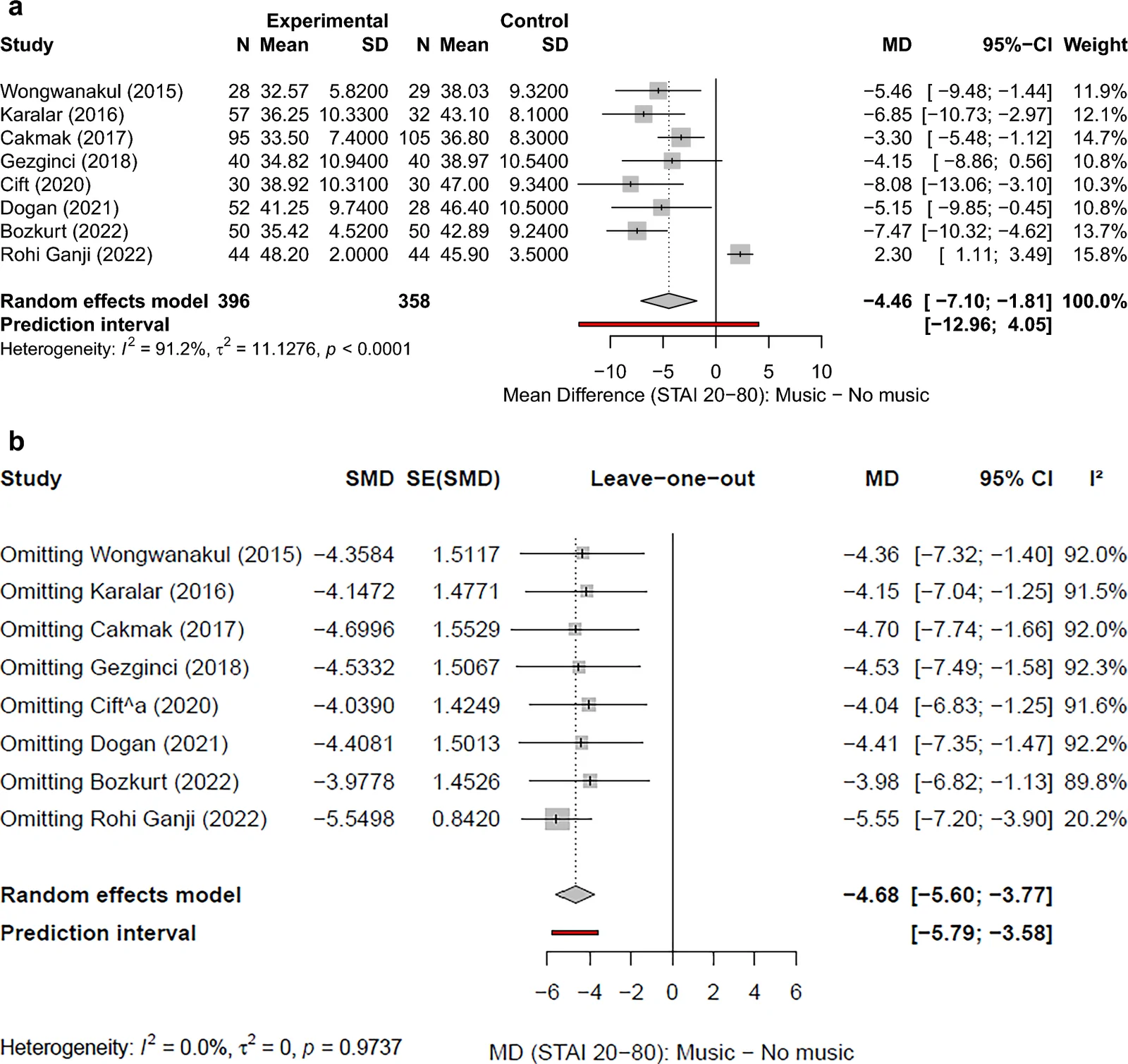

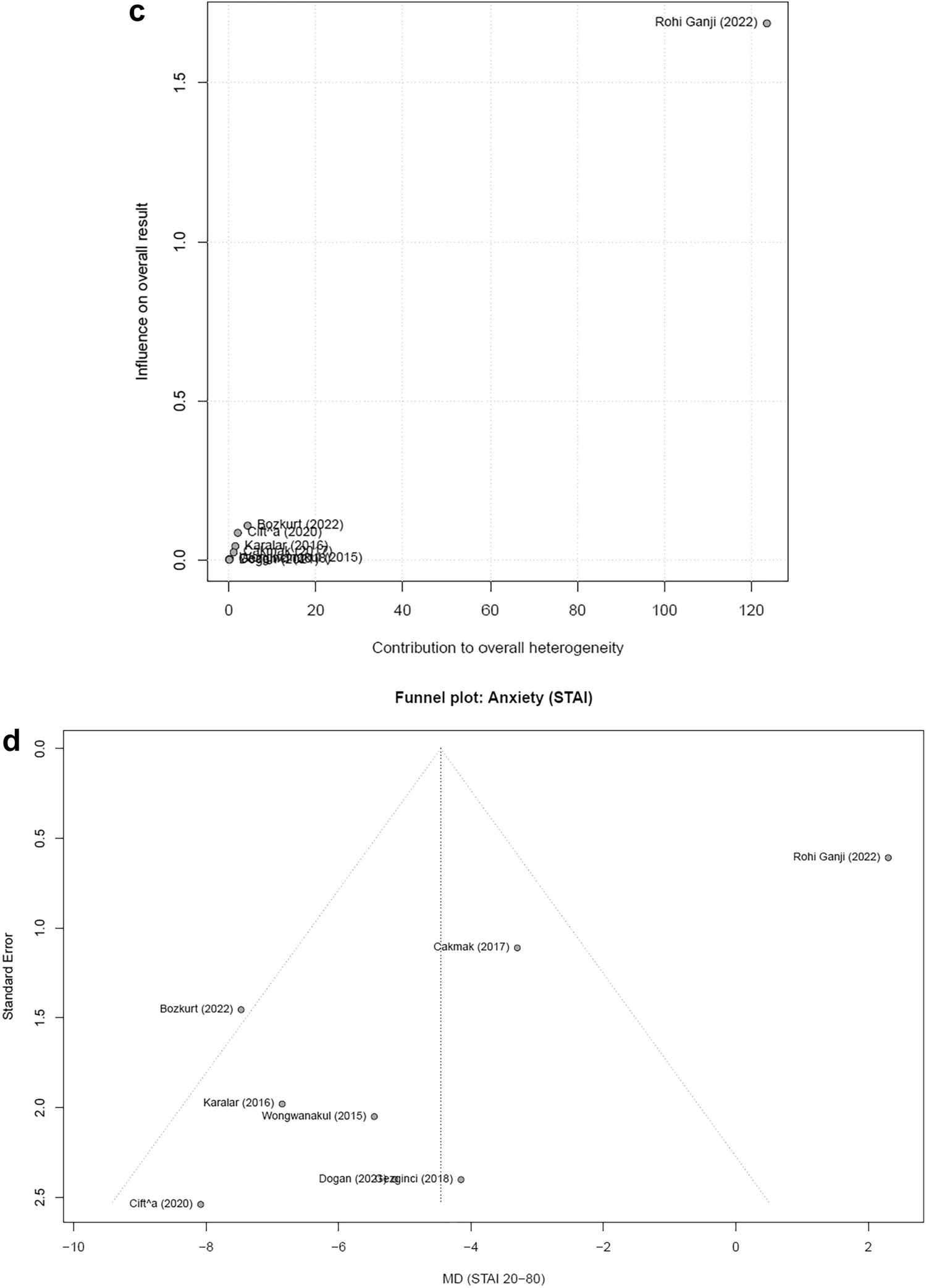
(a) Forest plot of anxiety measured by the State-Trait Anxiety Inventory (STAI) comparing Music versus Control groups, (b) Leave-one-out sensitivity analysis for heterogeneity assessment, (c) Baujat plot identifying studies contributing most strongly to the overall heterogeneity and pooled effect, and (d) Funnel plot assessing publication bias among the included studies.

Eight studies were included in the meta-analysis of anxiety measured using STAI. Music was associated with significantly lower STAI scores compared with the control condition (MD –4.46; 95% CI –7.10 to –1.81; *p* < 0.01). Considerable heterogeneity was observed across studies (*I*^2^ = 91.2%) (Fig. 3a).

The leave-one-out sensitivity analysis showed that the direction of the pooled estimate remained favorable to music after the sequential exclusion of each individual study (Fig. 3b). However, the magnitude of the pooled effect varied across iterations, and substantial heterogeneity persisted, indicating that the variability could not be attributed to a single influential study.

The Baujat plot identified studies with relatively greater contributions to heterogeneity, although no individual study completely explained the observed inconsistency (Fig. 3c).

Visual inspection of the funnel plot suggested possible asymmetry (Fig. 3d). Nevertheless, because only eight studies were available, formal regression-based tests of funnel plot asymmetry were not considered sufficiently reliable. Therefore, publication bias or other small-study effects could neither be confirmed nor excluded.

### Exploration of heterogeneity

The substantial heterogeneity observed in both analyses was consistent with the clinical and methodological diversity of the included trials. Potential sources of variability included patient-selected versus investigator-selected music, differences in music genre, the use of conventional or noise-canceling headphones, delivery through room speakers, intervention timing, and differences in analgesic and sedative protocols.

The trials also differed in patient characteristics, stone location and burden, lithotripter technology, and the methods used to administer and monitor the music intervention. These factors may have modified both baseline anxiety and the magnitude of the response to music.

Formal subgroup analyses were limited by the small number of studies within each category, incomplete reporting of relevant characteristics, and overlap between intervention features. Consequently, reliable conclusions regarding individual effect modifiers could not be established, and the sources of heterogeneity were interpreted primarily through descriptive comparisons of study characteristics.

### Risk of bias

All included studies were randomized controlled trials and were assessed using the Cochrane RoB 2 tool. One study was judged to have an overall low risk of bias, while the remaining 11 studies were classified as raising some concerns. No study was judged to have an overall high risk of bias.

Concerns most frequently arose in the measurement-of-outcome domain. Anxiety was assessed using patient-reported instruments, and participants could not be blinded to the music intervention. Knowledge of group allocation may, therefore, have influenced subjective outcome reporting.

**Fig. 4:**
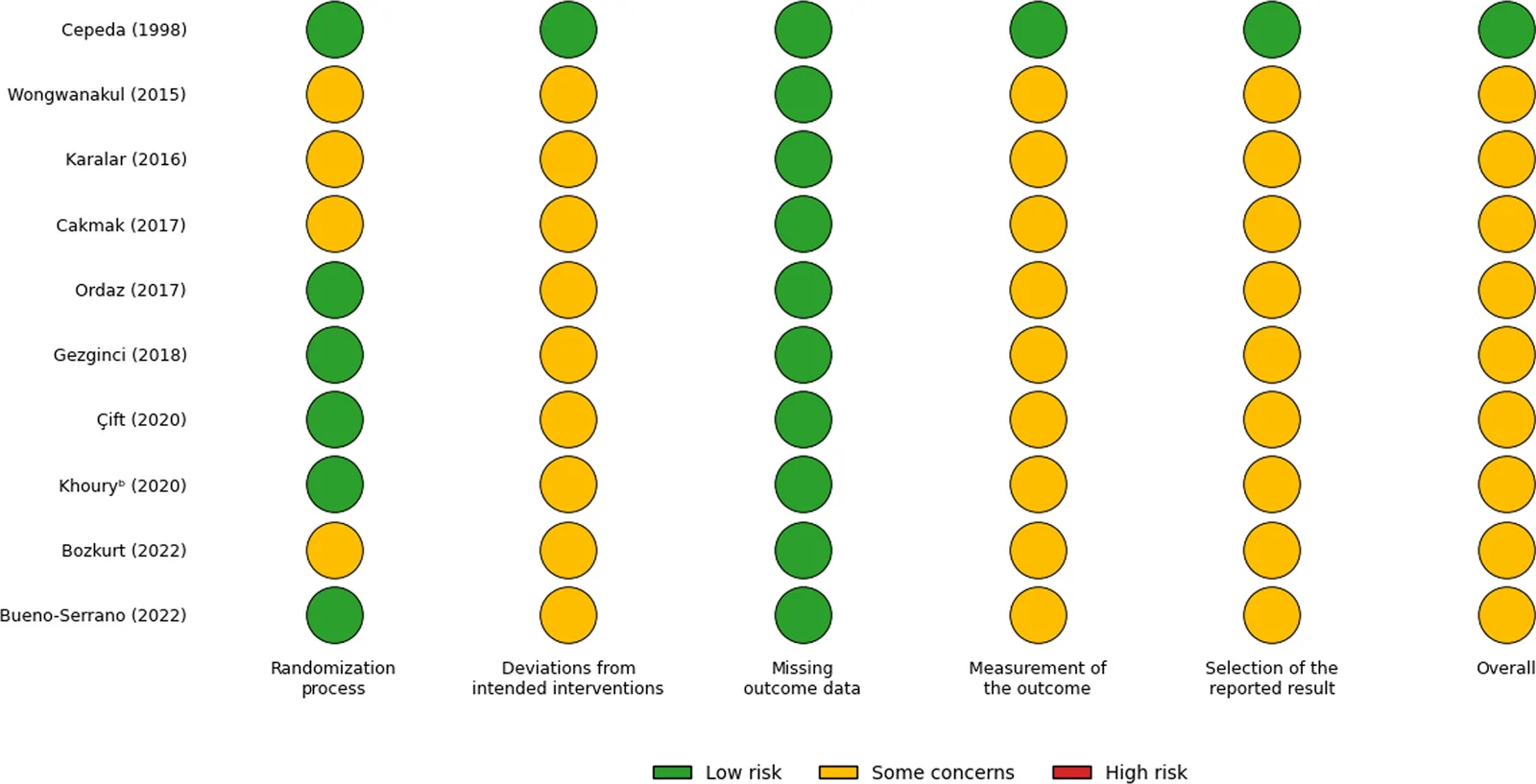
Risk of bias assessment of the included randomized controlled trials using the Cochrane Risk of Bias 2 (RoB 2) tool.

The selection-of-reported-result domain also frequently raised some concerns because pre-specified protocols or detailed statistical analysis plans were unavailable or insufficiently reported in several studies. In contrast, the risk of bias due to missing outcome data was generally low, as most trials reported no substantial losses to follow-up. The traffic-light plot summarizing the RoB 2 assessment is presented in Fig. 4.

### Trial sequential analysis

The TSA was performed to evaluate the robustness of the pooled evidence and the risk of random errors. For both anxiety outcomes (VAS and STAI), the cumulative Z-curve crossed the conventional significance boundary and surpassed the required information size, indicating that the available evidence is sufficient to confirm a statistically significant benefit of music intervention. (See Supplementary Figs. 1, 2).

Under the pre-specified assumptions, the TSA findings suggest that the statistically significant pooled effects were unlikely to be explained exclusively by sparse data or repeated significance testing. Nevertheless, TSA does not account for risk of bias, clinical heterogeneity, selective outcome reporting, or publication bias and should therefore be interpreted together with the conventional meta-analysis.

## Discussion

This systematic review and meta-analysis found that music was associated with lower anxiety scores among patients undergoing ESWL. The pooled reduction was approximately 1.0 point on the VAS and 4.5 points on the STAI. However, both analyses were characterized by substantial or considerable heterogeneity, and the certainty of evidence was very low.

Our findings are generally consistent with earlier evidence but provide a more anxiety-focused and updated assessment. Wang et al. included five randomized trials and reported lower anxiety and pain with music [8]. Hu et al. evaluated music and audiovisual distraction and found a reduction in anxiety, but emphasized weak evidence, substantial heterogeneity, and uncertainty regarding clinical importance [9]. Lei et al. included ten trials and reported beneficial effects of music, although their primary focus was analgesia rather than anxiety [10].

The present review included 12 randomized trials and incorporated studies published after the searches of previous reviews, including Dogan et al. and Rohi Ganji et al. [25, 28]. In addition, we analyzed VAS and STAI outcomes separately, used the contemporary RoB 2 framework, and applied TSA to assess the potential influence of random error.

The statistical effects should not automatically be interpreted as clinically important. An approximately one-point reduction on an anxiety VAS and a 4.5-point reduction on the STAI may represent a modest average change. A minimal clinically important difference has not been specifically established for anxiety during ESWL, and patient-level responses may vary. Therefore, the findings demonstrate a statistical association but do not establish that all patients experience a perceptible or meaningful improvement.

The substantial heterogeneity is central to the interpretation of this review. Music interventions varied in genre, patient choice, duration, timing, volume, delivery method, and use of noise-canceling technology. Some interventions may have functioned primarily as auditory distraction, while others may have had emotional or relaxation-related effects.

The comparison between patient-selected and investigator-selected music may be particularly relevant. Familiar or preferred music may provide greater emotional engagement, whereas standardized classical or instrumental music may be easier to reproduce but less personally meaningful. Nevertheless, the available trials were too few and too heterogeneous to establish whether patient choice modifies the intervention effect.

The mode of delivery may also influence outcomes. Most studies used headphones, but only some used noise-canceling technology. Headphones can improve auditory focus and reduce exposure to environmental sounds, whereas active noise cancelation may have an independent effect by attenuating the repetitive sound of the lithotripter. Conversely, music played through room speakers does not isolate the patient from procedural noise and may have a different effect.

Differences in pharmacological management constitute another important potential source of heterogeneity. Some studies used no routine analgesia, whereas others administered opioids, non-steroidal anti-inflammatory drugs, sedatives, or patient-controlled analgesia. These medications may directly influence anxiety, discomfort, alertness, and subjective reporting. Consequently, the pooled estimate represents an average across substantially different peri-procedural care pathways and should not be assumed to apply uniformly to every ESWL setting.

Patient populations and procedural characteristics also differed. Trials included renal stones, ureteral stones, or both, and used different electrohydraulic and electromagnetic lithotripters. Differences in baseline anxiety, previous ESWL exposure, stone burden, shock-wave intensity, procedure duration, and cultural familiarity with the music may have further contributed to the observed variability.

Leave-one-out analyses showed that no single trial was solely responsible for the direction of the pooled estimates. This supports the numerical stability of the average association but should not be interpreted as resolving heterogeneity. Sensitivity analysis cannot eliminate limitations related to subjective outcomes, incomplete blinding, variation in co-interventions, or selective reporting.

Possible funnel plot asymmetry provides an additional reason for caution. With only ten studies in the VAS analysis and eight in the STAI analysis, statistical methods for detecting asymmetry have limited power. Moreover, heterogeneity itself may produce an asymmetric funnel plot. Therefore, publication bias cannot be confirmed or excluded.

TSA suggested that the cumulative evidence crossed the relevant statistical monitoring boundaries under the pre-specified assumptions. This finding reduces the likelihood that conventional significance resulted exclusively from sparse data and repeated testing. However, TSA primarily addresses random error. It cannot compensate for systematic bias, clinical heterogeneity, selective reporting, or low methodological quality.

Music nevertheless has practical advantages. It is inexpensive, non-invasive, relatively easy to implement, and can usually be incorporated without changing the technical conduct of ESWL. No pharmacological monitoring is required for the music intervention itself. However, its use should be considered an optional adjunct rather than a replacement for appropriate counseling, analgesia, sedation, or individualized procedural care.

Future trials should use standardized and transparently reported music protocols, distinguish patient-selected from investigator-selected music, report the use of noise-canceling headphones, and control or stratify for analgesia and sedation. Baseline and post-procedural anxiety should be measured using validated instruments, and investigators should report both mean effects and the proportion of patients achieving a clinically meaningful improvement.

### Strengths and limitations

This review has several strengths. It was restricted to randomized controlled trials, included an updated search through March 2026, focused specifically on anxiety, analyzed VAS and STAI separately, applied RoB 2, and incorporated sensitivity, influence, and TSA methods.

Several limitations must also be acknowledged. First, substantial heterogeneity limits the generalizability of the pooled estimates. Second, most outcomes were subjective, and participant blinding was not feasible. Third, music type, selection, timing, delivery, and duration were not standardized. Fourth, analgesic and sedative protocols varied substantially.

Fifth, the small number of studies within clinically relevant categories prevented reliable subgroup meta-analysis. Sixth, small-study effects and publication bias could not be reliably evaluated. Seventh, clinically important thresholds specific to ESWL-related anxiety are unavailable. Finally, TSA findings depend on the anticipated intervention effects, variance, diversity estimates, and monitoring boundaries selected for the analysis.

## Conclusion

Music was associated with lower VAS and STAI anxiety scores during ESWL and may be considered a feasible non-pharmacological adjunct to standard peri-procedural care. However, substantial heterogeneity, variability in music and analgesia protocols, risk-of-bias concerns, possible small-study effects, and very low certainty of evidence limit confidence in the magnitude and clinical importance of the pooled effects.

Further adequately powered and prospectively registered randomized trials using standardized interventions and clinically meaningful anxiety outcomes are needed to clarify which patients are most likely to benefit and how music should be implemented during ESWL.

## Supplementary Information

Below is the link to the electronic supplementary material.

Supplementary file1 (DOCX 255 KB)

## Data Availability

No datasets were generated or analysed during the current study.

## Abbreviations

ESWL: Extracorporeal Shock Wave Lithotripsy
VAS: Visual Analog Scale
STAI: State-Trait Anxiety Inventory
MD: Mean Difference
CI: Confidence Interval
PRISMA: Preferred Reporting Items for Systematic Reviews and Meta-Analyses
MEDLINE: Medical Literature Analysis and Retrieval System Online
MeSH: Medical Subject Headings
RCTs: Randomized Controlled Trials
RCT: Randomized Controlled Trial
RoB 2: Risk of Bias 2
I2: Higgins’ I-squared statistic
TSA: Trial Sequential Analysis
α: Alpha error
β: Beta error
RIS: Required Information Size
D2: Diversity-adjusted information size / diversity statistic
SMD: Standardized Mean Difference
PCA: Patient-Controlled Analgesia
PMCID: PubMed Central Identifier
PMID: PubMed Identifier
FL: Florida
KS: Kansas
SP: São Paulo
USA: United States of America
