# Supplementary material for: Music as a non-pharmacological intervention for anxiety during extracorporeal shock wave lithotripsy: a systematic review, meta-analysis, and trial sequential analysis of randomized controlled trials
Source: Int Urol Nephrol. 2026 Jul 21;58(12):5317–30. doi: 10.1007/s11255-026-05266-2 (PMC13768247; doi:10.1007/s11255-026-05266-2)
Supplement: Supplementary file 1 [file 11255_2026_5266_MOESM1_ESM.docx]

Supplementary Figure 1. Trial sequential analysis for VAS.

Supplementary Figure 2. Trial sequential analysis for STAI.
